# The Amsterdam Studies of Acute Psychiatry - II (ASAP-II): a comparative study of psychiatric intensive care units in the Netherlands

**DOI:** 10.1186/1471-2458-9-318

**Published:** 2009-09-03

**Authors:** Vincent Koppelmans, Robert Schoevers, Cecile Gijsbers van Wijk, Wijnand Mulder, Annett Hornbach, Emile Barkhof, André Klaassen, Marieke van Egmond, Janine van Venrooij, Yan Bijpost, Hans Nusselder, Marjan van Herrewaarden, Igor Maksimovic, Alexander Achilles, Jack Dekker

**Affiliations:** 1 Arkin Mental Health Care, Amsterdam, The Netherlands; 2 VU University Medical Centre, Department of Psychiatry, Amsterdam, The Netherlands; 3 inGeest Mental Health Care, Amsterdam, The Netherlands; 4 Kliniek voor Duurzaam Verblijf, Beilen, The Netherlands; 5 inGeest Mental Health Care, Zuiderpoort, Haarlem, The Netherlands; 6 Academic Medical Centre/De Meren Mental Health Care, Amsterdam, The Netherlands; 7 Dijk & Duin Mental Health Care, Castricum, The Netherlands; 8 Dimence Mental Health Care, Deventer, The Netherlands; 9 inGeest Mental Health Care, Spaarnepoort, Hoofddorp, the Netherlands; 10 Arkin Mental Health Care, SPDC Noord, Amsterdam, The Netherlands; 11 Arkin Mental Health Care, SPDC COW, Amsterdam, The Netherlands; 12 inGeest Mental Health Care, Valeriuskliniek, Amsterdam, The Netherlands; 13 Arkin Mental Health Care, SPDC Oost, Amsterdam, The Netherlands; 14 VU University, Faculty of Psychology and Pedagogy, Department of Clinical Psychology, Amsterdam, The Netherlands

## Abstract

**Background:**

The number of patients in whom mental illness progresses to stages in which acute, and often forced treatment is warranted, is on the increase across Europe. As a consequence, more patients are involuntarily admitted to Psychiatric Intensive Care Units (PICU). From several studies and reports it has become evident that important dissimilarities exist between PICU's. The current study seeks to describe organisational as well as clinical and patient related factors across ten PICU's in and outside the Amsterdam region, adjusted for or stratified by level of urbanization.

**Method/Design:**

This paper describes the design of the Amsterdam Studies of Acute Psychiatry II (ASAP-II). This study is a prospective observational cohort study comparing PICU's in and outside the Amsterdam region on various patient characteristics, treatment aspects and recovery related variables. Dissimilarities were measured by means of collecting standardized forms which were filled out in the framework of care as usual, by means of questionnaires filled out by mental health care professionals and by means of extracting data from patient files for every consecutive patient admitted at participating PICU's during a specific time period. Urbanization levels for every PICU were calculated conform procedures as proposed by the Dutch Central Bureau for Statistics (CBS).

**Discussion:**

The current study may provide a deeper understanding of the differences between psychiatric intensive care units that can be used to promote best practice and benchmarking procedures, and thus improve the standard of care.

## Background

The number of patients in whom mental illness progresses to stages in which acute, and often forced treatment is warranted, is on the increase across Europe. The overall number of involuntary admissions has increased in countries such as Germany, France, England, Austria, Sweden and Finland [1-3]. In the Netherlands, the number of compulsory admissions has doubled between 1979 and 2004, rising from 23 to over 53 per 100.000 inhabitants [4]. This increase includes both compulsory admissions in crisis situations without reference to the courts ("compulsory admissions") and compulsory admissions after recourse to the courts ("court orders") [4]. In the Amsterdam area, the number of compulsory admissions even rose by 319% to 86 per 100,000 in the period between 1979 and 2004 [5].

As a consequence, more patients are involuntarily admitted to Psychiatric Intensive Care Units (PICU). The proportion of coercive admissions to Amsterdam Psychiatric Intensive Care Units is now as high as 80% [6].

PICU's, defined as units providing assessment, care and short-term intensive treatment for acutely disturbed psychiatric patients who cannot be dealt with on regular open wards [7,8] have been criticized for a poor environment such as deteriorated rooms and furniture and limitations for patient activities, such as fresh air and exercise, due to shortage of staff [9], high levels of coercion [10] and a lack of evidence of regular procedures from controlled trials [11,12]. As both mental health providers and governmental authorities are increasingly emphasising the client perspective, the interest into admission- and intervention policies of PICU's has increased over the last 15 years [12-14]. At the same time, management- and financial entities also stress the importance of cost-effectiveness and uniformity. Major goals are therefore to decrease the level of coercion and increase treatment quality as well as evaluating current policies [15] In the last decade, national organisations such as the Psychiatric Intensive Care Advisory Service (PICAS) and the National Association of Psychiatric Intensive Care Units (NAPICU) in the United Kingdom, have been developed to improve the standard of care delivered within PICU's. In 2002, Pereira and Clinton published a report on national minimum standards for general adult services in PICU's and low secure environments. This report provides, among others, guidelines regarding criteria for admission, core interventions, physical environment and personnel [16]. Kallert et al. [17] recently performed a European multi centre study to evaluate the levels of coercion in participating PICU's.

From several studies and reports it has become evident that important dissimilarities exist between PICU's in different countries in terms of patient selection, type and quality of care and treatment outcome [2,3,17,18]. Also within countries, PICU's may show significant variation. Often it is unknown whether such differences in structure and functioning of PICU's are based on clinical considerations or a result of historical or financial developments.

PICU's may differ in organizational structure as well as in treatment policies such as medication prescriptions, quantity of face-to-face time per patient, mean length of stay, use and duration of seclusion, implementation of legal measures, staffing, and availability of individualised treatment regimens [11,12,19-21]. In a similar way, patient groups may also differ between PICU's. In general, metropolitan areas accommodate higher percentages of ethnic minority and migrant populations, who may differ on characteristics such as psychiatric morbidity, socio-demographic variables, the size and structure of the social network and (prior) (co-morbid) substance abuse [22]. Differences in levels of urbanization may possibly account for differences in patient selection and psychiatric treatment between units. A number of studies have shown an increasing level of urbanization to be associated with higher incidence rates of psychosis [23-25] and other mental disorders such as substance abuse.

The above factors may influence the results of PICU admissions in terms of treatment outcome (degree of recovery of the psychiatric disorder). The question is which characteristics of the organizational structure, the treatment delivered, or the patient are stipulating for recovery. In a recent study, Wynaden et al. [26] stress the importance of ongoing evaluations of PICU patient populations to promote best practice initiatives in psychiatric care. Since the development of the first PICU's in the early 1970's [12], several studies have explored and compared PICU's on specific subjects such as patient characteristics and treatment outcome. Yet, to our knowledge no studies have investigated the broad range of all these variables and their mutual relationship using a comprehensive integrated model. Several studies investigated specific PICU characteristics such as bed numbers, staffing levels, admission criteria, physical environment, psychosocial methods and pharmacotherapy, but none of these managed to relate such characteristics to treatment outcome [12].

The current study seeks to describe organisational as well as clinical and patient related factors across 10 PICU's in and outside the Amsterdam region, providing also differences in the level of urbanization. The study is part of a larger project also involving a city wide study on factors leading to coercive admission (ASAP-I) [27].

The main research questions are: *1) Do the participating PICU's differ with respect to the type of patients they admit? 2) Do these PICU's differ in the way treatment and care are implemented? 3) What are the differences with regard to outcome at discharge? 4) Are these characteristics related to level of urbanization?*

## Methods/Design

This paper describes the design of the Amsterdam Studies of Acute Psychiatry II (ASAP-II). ASAP-II is a series of studies examining different aspects of psychiatric intensive care units. Before the main study was conducted, various descriptive statistics of every PICU were collected, such as number of admissions per year, security measures, ward characteristics (e.g. number of beds, number of seclusion rooms, recreational facilities), facilities available in the seclusion room and number of staff (full-time equivalents) categorized by discipline. Furthermore, PICU policies and characteristics of patients who had been admitted in the past year were studied and compared.

### Study design

This main study is a prospective observational cohort study.

### Participating centres

Three larger mental health care institutions cater for the acute admissions within the borders of the municipality of Amsterdam. Together they account for six PICU's providing all emergency inpatient psychiatric care in this area. Although each PICU has its own catchment area, patients are admitted to one of the other wards depending upon availability of beds. Within the Amsterdam area, the majority of the emergency psychiatric consultations are performed by the centralised Psychiatric Emergency Service Amsterdam (PESA). The majority of acutely admitted patients have had a psychiatric evaluation by PESA before being admitted to one of the PICU's. Compulsory admissions are distributed to the PICU corresponding with the patients abode directly, or indirectly through the Temporary Admission Unit (TAU). The TAU is a special PICU that was set up in 2001 to resolve a shortage of bed capacity in the city, and to reduce the period patients had to wait in police cells or crisis centres before being admitted to a psychiatric hospital. 80% of all the patients admitted at the TAU are being redirected to one of the five other PICU's within days [28,29]. In addition, all PICU's receive direct (compulsory or voluntary) admissions from service providers within their own catchment area during office hours.

All of the Amsterdam PICU's participate in the current study. In order to increase contrasts associated with level of urbanization, four PICU's located outside the city of Amsterdam are also included in this study. All but two of the participating wards have a psychiatric residency program. In these wards, residents treat patients under close supervision of the psychiatrist working on the ward.

### Level of urbanization

Two different indices for urbanization rate were computed for every PICU and the corresponding catchment area:

#### 1) Address Density Index (ADI)

The ADI concerns the number of addresses per squared kilometre and is a measure for human activity that takes into account all domestic, business, education and leisure addresses. For every municipality, an address-density index is published and updated every year by the Dutch Central Bureau for Statistics (CBS) [30]. For each PICU, numbers were derived from the CBS website for the year in which the data were collected.

To obtain the address density index per catchment area, the weighted mean address density index of the municipalities in the particular catchment area was calculated. The address density indices of the municipality were weighted by the number of inhabitants in order to adjust for the size of the municipality (table 1 - Urbanization data).

**Table 1:** Urbanization data

**Catchment Area/PICU**	**Municipality**	**Year of data collection**	**Inhabitants**	**ADI**	**ADI**	**CUI**	**CUI**	**CUI**	**CUI**	**CUI**
				**number**	**ctagorie**	% Very Strongly Urbanized*	% Strongly Urbanized*	% Moderlately Urbanized*	% Weakly Urbanized*	% Not Urbanized*
Amsterdam	Amsterdam	2005	742790	6051		82	16	2	0	1
	Amsterdam	2006	743070	6051		81	16	1	1	1
*OVERALL*					**Very strongly urbanized**	**81**	**16**	**2**	**1**	**1**

Castricum	Beemster	2005	8520	548		0	4	5	25	67
	Beverwijk	2005	36860	2363		53	31	6	0	9
	Castricum	2005	35100	1146		0	32	21	33	14
	Edam-Volendam	2005	28350	1413		0	48	40	11	1
	Heemskerk	2005	36440	1974		24	48	19	5	4
	Landsmeer	2005	10280	831		0	0	38	45	18
	Oostzaan	2005	9180	940		0	0	60	29	11
	Purmerend	2005	77070	2121		23	61	12	5	0
	Uitgeest	2005	11780	931		0	0	44	54	2
	Waterland	2005	17340	583		0	0	13	40	48
	Zaanstad	2005	139830	1764		19	37	25	12	6
	Zeevang	2005	6270	240		0	0	0	2	98
*OVERALL*					**Strongly urbanized**	**17**	**37**	**21**	**15**	**10**

Haarlem	Bennebroek	2006	5130	665		0	0	0	99	1
	Bloemendaal	2006	16980	1064		5	15	35	22	23
	Haarlem	2006	147010	3151		72	23	5	0	1
	Haarlemmerliede & Spaarnwoude	2006	5480	579		0	0	9	29	62
	Heemstede	2006	25660	1483		0	58	13	21	7
	Zandvoort	2006	16660	1408		0	58	15	18	9
*OVERALL*					**Very strongly urbanized**	**49**	**28**	**9**	**9**	**5**

Hoofddorp	Aalsmeer	2006	24150	665		0	0	9	63	28
	Amstelveen	2006	78770	2029		27	49	17	5	2
	Haarlemmermeer	2006	135140	1433		7	44	25	14	11
	Uithoorn	2006	26850	1339		0	41	33	13	13
*OVERALL*					**Strongly urbanized**	**11**	**41**	**22**	**15**	**10**

Deventer	Deventer	2005	95620	1690		19	31	27	11	13
	Olst-Wijhe	2005	17080	372		0	0	0	41	59
*OVERALL*					**Moderately urbanized**	**16**	**26**	**23**	**16**	**20**

* due to rounding, numbers might add up to over a 100%

The acquired numerical mean weighted ADI scores were then assigned to a categorical CBS standard classification (table 1 - Urbanization data & Table 2).

**Table 2:** Dutch Central Bureau for Statistics Address Density Index

**Level**	**Addresses per km2**	**Catagorie**
1	> 2500	Very strongly urbanized
2	1500 - 2500	Strongly urbanized
3	1000 - 1500	Moderately urbanized
4	500 - 1000	Weakly urbanized
5	< 500	Not urbanized

According to the CBS classification, the ADI of the catchment areas of the Amsterdam and Haarlem PICU's are classified as 'very strongly urbanized', the ADI of the PICU's of Castricum and Hoofddorp are 'strongly urbanized' and the ADI of the catchment area of the Deventer PICU is classified as 'moderately urbanized' (table 1 - Urbanization data).

#### 2) Categorical Urbanization Index (CUI)

The CUI is a 5-level categorical classification applied in the CBS standard (See Table 2). The five CUI levels are expressed in percentages, corresponding to the size of the municipality they represent. For every municipality, a CUI is published and updated every year by the CBS. Again, numbers per PICU were derived from the CBS website according to the year in which the data collection took place [30].

To obtain the categorical urbanization index per catchment area, the weighted mean categorical urbanization index levels of the municipalities in that particular catchment area were calculated. The CUI levels of each municipality were weighted by the number of inhabitants in order to correct for the size of the municipality (table 1 - Urbanization data).

**Figure 1:**
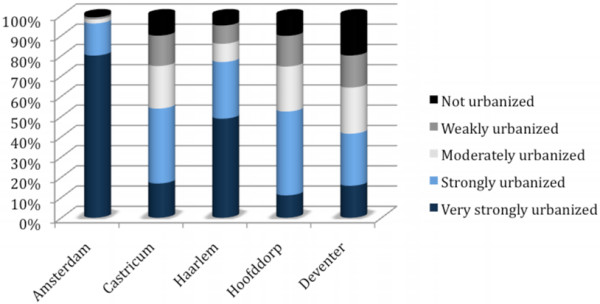
**Categorical Urbanization Index**.

Figure 1 shows that the ADI of the catchment area of the Amsterdam PICU's are overall considered very strongly urbanized, however, the more detailed CUI of the catchment areas shows that the very strongly urbanized areas are substantially smaller (See figure 1 and table 1 - Urbanization data).

##### Informed consent

The study concerns clinical data derived from case files used in daily practice. As the study concerned data used in daily health care and patients were not treated according a study protocol but received care as usual, signing an informed consent was not required according to the Medical Ethical Committee (MEC) based on the Dutch law on Medical Scientific Research involving persons (WMO).

Patient anonymity was guaranteed by means of storing patient data under a unique research number. The list containing the linked names and research numbers was only available to the primary investigator (VK) and the principal investigators (RS, JD, CGvW).

### Participants

It was estimated that in a period of three months, at least 40 eligible patients would be admitted per unit, and be included in this study. The inclusion period for each unit was therefore set at three months, or a minimum of 40 consecutively admitted patients. If less than 40 patients were included within three months, inclusion continued until the number of 40 had been reached. Patients were followed up until discharge. No further inclusion criteria were determined, and all admitted patients were included in the study. The overall inclusion period ran from 14-10-2005 until 19-06-06 across the participating centres. This spread in data collection was the result of limited capacity of research personnel responsible for collecting the actual data, which forced us to start data collection at the participating units consecutively rather than parallel.

### Measures

All of the study items were collected in a single Case Registration Form (CRF). Data were collected at three points in time: at admission (t^1^), during the PICU stay (t^2^) and at discharge (t^3^). The majority of data concerned objective quantitative patient and treatment characteristics extracted from (electronic) patient files (see Table 3 for more details).

**Table 3:** Assessments of the ASAP-II study

**Measure**			
	**t^1^**	**t^2^**	**t^3^**

*Questionnaire*			
REHAB (extent of deviant and common behavior)	**X**		**X**
M-BPRS (severity of psychiatric symptoms)	**X**		**X**

*Form*			
Article 38/Article 39		**X**	
Health Care Incident Report (HCIR)		**X**	
Illegitimate Absence Form (IAF)		**X**	

*Variable*			
date of birth	**X**		
gender	**X**		
type of admission	**X**		
referral characteristics	**X**		
social situation	**X**		
ethnic background	**X**		
socioeconomic status	**X**		
criteria for admission	**X**		
social network	**X**		
medication use prior to admission	**X**		
substance abuse	**X**		
somatic comorbidity	**X**		
DSM-IV TR diagnosis	**X**		**X**
medication prescriptions		**X**	**X**
seclusion related variables		**X**	
therapy		**X**	
length of stay			**X**
discharge variables			**X**
perspectives			**X**

Diversity between psychiatric intensive care units was measured on the level of patient characteristics, treatment characteristics, and treatment outcome.

#### 1) Patient characteristics

Patient characteristics regard differences between patients associated with socio-demographic and admission criteria:

- date of birth

- gender

- admission characteristics: time and date of admission; whether it was a first admission; voluntary or compulsory; planned or acute admission; name of resident or psychiatrist admitting the patient.

- referral characteristics: referring health care professional or institution.

- social situation: relational status, habitual status, care for children.

- ethnic background: country of origin from patient, mother and father; legal status

- socio-economic status: source of income; occupation; income; level of education

- previous history; number and type of prior voluntary and involuntary admissions to PICU's or other mental health care institutions; time since last contact with mental health care.

- social network: contact with parents/children/partner/siblings.

- medication use prior to admission: type and dosages

- substance abuse at admission: type of substance; urine control and results if available

- physical health status: somatic comorbidities; physical examination; need for medical treatment or nursing; need for medication; need for specialist consultation;

- DSM-IV TR diagnosis: clinical diagnosis according to the treating psychiatrist or resident classified according to the DSM-IV

- severity of psychiatric symptoms: as measured by the M-BPRS

- extent of deviant and common behaviour: as measured by the REHAB

#### 2) Treatment received

This concerns different aspects of psychiatric treatment and care provided during admission and comprises:

- Medication prescriptions: (generic)name; medication doses; route of administration (oral/intramuscular/other)

- Implementation of coercive measures: starting date and termination of coercive measures according to Article 38 and Article 39 of the Netherlands law governing involuntary Admissions to Psychiatric Institutions. Coercive measures include seclusion, separation, fixation, forced medication and forced nutrition or food. In line with legal regulations two further facts were noted: a) whether patients actively opposed the use of coercive measures; b) whether alternative and less coercive measures have been tried to cope with the situation.

- Seclusion characteristics: time, date and duration of each seclusion were noted, as well as the motivation for seclusion and the discipline of the health care professional who initiated the seclusion. In emergency situations, seclusion may be initiated by mental health nurses but the responsible physician has to be alerted immediately.

- treatment: time, date and duration of the contact with a health care professional; discipline of the professional (psychiatrist/resident/nurse/...)

- length of stay in days

#### 3) Recovery

Recovery regards the extent of psychiatric stabilization during admission. This is appraised in several ways.

- By clinical judgment of the treating resident or psychiatrist and the community mental health nurse or case manager.

- Discharge variables: judicial status at discharge; motivation for discharge, subsequent treatment; abode after discharge

- Medication prescriptions: (generic)name; medication doses; route of administration (oral/intramuscular/other)

- DSM-IV TR diagnosis at discharge (clinical diagnosis)

- Symptom severity: M-BPRS at discharge

- Extent of deviant and common behaviour: REHAB score at discharge

##### Measurement instruments

###### M-BPRS

The Dutch version [31] of the Modified Brief Psychiatric Rating Scale (Bigelow L, Murphy DL.: Guidelines and Anchor Points for Modified BPRS, submitted) [32] is a questionnaire which measures (the severity of) psychiatric symptoms. It was derived from the Brief Psychiatric Rating Scale developed in the late 1960s [33]. The M-BPRS is an observation list, covering the following 5 subscales: depression/anxiety, anergy, thought disturbance, activation/mania and hostility/suspiciousness. An overall score can be derived from these 5 subscales. The questionnaire consists of 27 items with scores on a 6-point Likert scale. The M-BPRS is administered by the treating psychiatrist or resident. Administration takes approximately 10 minutes or less. Reliability and validity of the BPRS were shown to be good [34].

###### REHAB

The Dutch version [35] of the Rehabilitation Evaluation Hall And Baker [36] measures common and deviant behaviour. The REHAB can be divided into two main parts: deviant behaviour (DB) and common behaviour (CB). The latter can be divided into three subscales: social activity (SA), speech skill (SS), and self-care (SC). An overall score 'common behaviour' can thus be derived. Social activity concerns the extent to which the patient engages in social activities. The subscale language disorder regards speech fluency, meaningfulness and clearness. Self-care bears upon the quantity of (bodily) care for self, shown by the patient. DB consists of seven items with scores on a 3-point Likert scale. The 16 CB items are scored on a horizontal line by which the severity of the aberration can be indicated by drawing a mark perpendicular on the horizontal line. The far left side of the line corresponds with absence of problems and runs to the far right side of the line, which corresponds with the utmost extent of aberration. A scoring template can be used to convert the position of the mark to a score, ranging form 0 till 9. Completion of the REHAB does not require specific experience with mental health care. Nevertheless, it has to be done by someone closely involved with the patient such as the community psychiatric nurse, the case manager, or a family member who is in frequent contact with the patient. Completion takes approximately 10 minutes. Reliability of the Dutch form was shown to be satisfactory, the validity was good [37].

##### (Legal) Forms

###### Article 38 and Article 39

Article 38 and Article 39 are segments of the Dutch law governing Coerced Admissions to Psychiatric Institutions regulating the following types of treatment: seclusion, separation, fixation, medication and food and nutrition. Article 39 concerns emergency situations that warrant immediate interventions. Article 38 bears upon planned and arranged care against the will of the patient. Moreover, coercion under Article 39 can only be lawfully applied for seven succeeding days. If coercive treatment is still considered necessary after these seven days, Article 38 states that all coercive measures should be specifically incorporated into the treatment plan, and an official request for them has to be granted to the medical superintendent before further application is justified.

In the Netherlands, it is obligatory to register the application of coercive measures by filling out distinct standard forms for Article 38 and Article 39. These forms request patient characteristics and data about the emergency situation that justified the use of forced treatment. Subsequently, it is required to state what alternative measures have been applied in order to prevent the use of coercion, and whether or not an official protest was made against this measure by the patient or his representative. For both Article 38 and 39, a legal form must be filled out at the start and termination of the applied measure. These forms are to be sent to the Netherlands Health Care Inspectorate to ensure care providers and institutions comply with laws and regulations.

###### Health Care Incident Report (HCIR)

The HCIR form is designed to improve the quality of care by systematically reporting and analysing incidents in patient care. Incidents are divided into several main classes, the majority of which bear upon aggression, falling, faults in the delivery of medication, sexual harassment and discrimination. The form captures data about the sort of incident, the location where- and the circumstances in which the incident took place.

###### Illegitimate Absence Form

In case of illegal absence it is required that a form is filled out describing the escape- (and return) date of the patient from (to) the PICU. From this, the period in which a patient has been illegally absent from the closed ward can be determined.

### Procedure

At the onset of the project, every participating unit received a number of M-BPRS and REHAB instruction booklets to support the raters in filling out the forms and to insure a standardized procedure.

Variables regarding t^1 ^(see Table 3) were collected at admission. The M-BPRS was filled out by the treating physician. The REHAB questionnaire was filled out by the community psychiatric nurse/case manager. The remaining information collected at t^1 ^was gathered by the research assistant (RA) who made weekly visits to all participating units. The RA compiled the patient characteristics by making use of the (electronic) patient file and by interviewing staff workers. In the same way, data were collected during t^2 ^(see Table 3). After the patients discharge, the RA collected the data regarding t^3 ^(see Table 3). The follow-up measurements of the M-BPRS and REHAB were again filled out by the responsible staff members.

### Data processing

The paper CRF files were entered and stored in a custom DOS-based electronic database program developed at Arkin Mental Health Care.

The variable 'ethnicity' was recoded in order to group patients into natives and immigrants: First generation immigrants were defined as patients who were born outside the Netherlands. Second generation immigrants were defined as persons who were born in the Netherlands themselves having at least have one parent who was born outside the Netherlands. A patient was only considered a native when he/she and both his/her parents were born in the Netherlands.

### Statistical analysis

M-BPRS scores and REHAB scores were divided into subscales in concordance with Lachar et al. and Van der Gaag and Wilken [34,35] respectively. Differences between scale data are examined using analysis of (co)variance (ANOVA/ANCOVA) and multiple analysis of (co)variance (MANOVA/MANCOVA). Post-hoc test with Bonferoni correction will be used to verify whether- and which significant differences exist between specific units.

Regarding variables with ordinal and nominal data, differences between units will be examined using cross tabulations and tested for statistical significance by means of Chi-square tests. Subsequently, in order to compare variables between units in which co-varying factors play a part, hierarchical regression analysis will be used.

All results will be calculated at a significance threshold of p < 0.05.

Comparisons between units are based on either the mental health care institution they are part of, or on level of urbanicity.

## Discussion

The current study seeks to provide information necessary to compare the functioning and outcome of psychiatric intensive care units located in different areas of the Netherlands. The study contains a comprehensive set of characteristics of patients and procedures regularly collected in mental health care. Comparing psychiatric intensive care units may provide us with new insights that can be used to promote best practice and benchmarking procedures, and thus improve the standard of care. However, there are pregnant limitations to this study that need to be addressed here. A preliminary reflection on the limitations and strengths of our design:

### Limitations of this design

#### Design

A first limitation is inherent to the pragmatic and descriptive design of this study and concerns the fact that the setup of this study is not a randomized controlled trial but a prospective observational cohort study which limits the possibility to infer causal relationships between variables and study outcomes due to confounding factors. Associations found in this study will therefore be mainly indicative. Nevertheless, this is the first study that takes into account a comprehensive set of variables relevant for emergency mental health care. The current study thus may provide a deeper understanding of the differences between psychiatric intensive care units.

Although the overall number of the study population is sufficiently large to be able to detect the hypothesised differences based upon the power calculation, a second limitation is that the subject numbers per PICU are relatively small given the number of variables taken into account. Nevertheless, the number do allow for multivariate analyses encompassing the primary variables from each of the relevant domains.

#### Data collection

A third limitation is that there were slight differences in the way some of the study variables were collected in the patient files of different institutions. At the time the data were collected, some units already used electronic patient files, whereas others still applied handwritten patient files. In two of the participating units, information on earlier treatment history could not always be obtained because of a switch to electronic patient files right before the onset of this study. Similarly, in some patients, information on previous treatment history was incomplete in terms numbers of former admissions, date of last admission since current admission, and information regarding type and date of last ambulatory treatment contact. However, all centres collected largely the same data and missing data could usually be manually retrieved from the patient files and recoded into the study variables. Preliminary results show that the core variables such as patient and discharge characteristics have nearly always been retrieved.

A fourth limitation concerns the fact that REHAB and M-BPRS questionnaires were collected as part of daily practice. Although instruction on the correct use of these instruments was regularly provided and available in the study forms, clinicians were not specifically rated in terms of inter-rater reliability. Furthermore, as the length of stay in PICU's is generally short and the population fluctuating, discharge characteristics were not always assessed on the exact moment of transmission to another ward of discharge, but could be filled out some days later. Still, study coordinators and supervisors made every effort to ensure that data were assessed directly at discharge to assure standardized objective measurements.

A fifth limitation may regard the fact that, for pragmatic reasons, the data collection started at one of the Amsterdam PICU's in October 2005, while the last episode of data collection started in March 2006 at the PICU of Hoofddorp. However, we are not aware of any changes in patient-, ward-, or contextual factors that may have systematically affected this data.

### Strong aspects of this design

The current study is one of very few multi centre studies in which a wide variety of longitudinal and cross-sectional *patient *data are systematically gathered in combination with important contextual variables concerning treatment and facilities. Although a few studies have investigated differences in characteristics of PICU's, such as number of beds, staffing and admission policy and treatment [7,38-40], most studies have not been able to relate these to patient characteristics and treatment outcome. To our knowledge, only one study, by Kallert et al. [17] has used a design in which a broad range of variables, in this particular case mostly related to coercion, were examined in different PICUs throughout Europe. However, Kallert et al. only included patients who actually experienced coercive measures during involuntary hospital admission and voluntary patients who reported at least three out of the five questions of the Perceived Coercion Scale from the MacArthur Admission Experience Survey, whereas the current study includes all the consecutive patients admitted to the participating wards during the inclusion period. Therefore, results from this study may be generalized to the whole PICU population.

Another strength of this study is the possibility to differentiate the characteristics according to level of urbanicity of the participating PICUs. This adds an extra dimension to the model that allows the study of the association between the metropolitan environment, patient characteristics and treatment outcome. Although it is known that metropolitan areas may contain a somewhat more complex patient population in terms of ethnicity and (comorbid) psychiatric and social problems [22], such a gradient has not been demonstrated for patients admitted to PICUs. We hypothesize that patients admitted in more urbanized areas show more severe of chronic mental illness, and higher levels comorbid conditions such as substance abuse. Getting to know different patterns and presentations of specific patient groups using psychiatric intensive care units may result in developing early and more specific interventions to reduce the length of stay and to improve adequate and timely care in a highly urbanized context.

This study provides a cross section of all acute psychiatric admissions within the city of Amsterdam. Because all psychiatric intensive care units from the Amsterdam region participate in this project, the outcome can be considered unbiased. Hence this study is representative for the catchment area of the total mental health care system covering the whole municipality of Amsterdam.

### Data security

Confidential information and participant names are secured by the medical confidentiality rules and are treated according to the code of conduct for medical research, developed by the FMWV (the Federation of Biomedical Scientific Societies).

The results of the participant questionnaires are not accessible to Mental Health workers. All study related documents and data are stored on a protected central server from the research department of Arkin Mental Health Care Amsterdam. Only members of the study have access to the respective files.

## Competing interests

The authors declare that they have no competing interests.

## Authors' contributions

VK was the primary investigator for this study. CGvW, WM, JD and RS are accountable for the study design and the conceptualization of this study. AH, EB, AK, MvE, JvV, YB, HN, MvH, IM and AA were responsible for the practical implementation of the study at the participating PICU's. VK, RS and JD participated in the draft manuscript of the proposal. JD and RS are responsible for the overall supervision of the ASAP studies. All authors have read and approved the final manuscript

## Pre-publication history

The pre-publication history for this paper can be accessed here:


